# A novel hybrid deep learning framework for customer churn prediction using RFM and embedding clustering

**DOI:** 10.1038/s41598-026-53220-0

**Published:** 2026-05-28

**Authors:** Samia Ibrahim, BenBella S. Tawfik, Mohamed Abdallah Makhlouf, B. Hafiz

**Affiliations:** Information System Department, Faculty of Computers and Informatics, Suez Canal University, Ismailia, Egypt

**Keywords:** Customer churn, Deep embedded clustering, RFM features, LSTM, GRU, Customer behavior analysis, E-commerce, Machine learning, Engineering, Mathematics and computing

## Abstract

Customer churn prediction in E-commerce remains a challenging task due to the lack of labeled data, and the limited ability of traditional machine learning models to capture complex and dynamic customer behavior patterns. Existing approaches either rely on handcrafted features without effective representation learning or apply deep learning models without incorporating meaningful customer segmentation. To address these limitations, this study proposes a unified hybrid framework that integrates RFM-based feature engineering, Deep Embedded Clustering (DEC), and deep learning models for joint customer segmentation and churn prediction. The proposed framework first learns compact latent representations using a deep autoencoder, followed by clustering customers into behaviorally meaningful groups using an improved DEC mechanism with validated cluster selection and refinement. These learned representations are then used as input to Gated Recurrent Unit (GRU) and Long Short-Term Memory (LSTM) models for multi-class churn prediction. The framework is evaluated on two real-world datasets, namely the Online Retail dataset and an Events dataset, which differ significantly in scale and behavioral complexity. Experimental results demonstrate that LSTM achieves an accuracy of 99.65% on the Online Retail dataset and 99.83% on the Events dataset, while GRU achieves 99.77% and 99.75%, respectively. Although traditional models such as Logistic Regression and Support Vector Machine achieve competitive performance, they show limited adaptability across heterogeneous data distributions. The results confirm that integrating representation learning with clustering and deep sequential models significantly enhances churn prediction performance. The proposed framework effectively transforms raw transactional data into structured, actionable insights that support customer retention strategies in E-commerce environments.

## Introduction

Customer churn refers to the proportion of customers who discontinue purchasing or interacting with a company^1,2^.In E-commerce environments, churn is considered one of the most critical performance indicators, as a high churn rate directly reflects weaknesses in customer satisfaction, service quality, or product experience. Beyond its direct financial impact, churned customers may also negatively influence brand reputation and reduce long-term customer acquisition efficiency. Therefore, understanding the underlying behavioral factors that lead to customer attrition is essential for developing effective and proactive retention strategies.

Customer segmentation is a fundamental component in addressing this challenge, as it enables organizations to group customers based on shared behavioral, demographic, and transactional characteristics^3^. Among various segmentation techniques, RFM (Recency, Frequency, Monetary value) analysis is widely adopted in customer analytics due to its simplicity and effectiveness^4^. It captures three essential behavioral dimensions: how recently a customer has purchased, how frequently they transact, and how much revenue they generate. This allows businesses to identify meaningful customer segments and design targeted marketing strategies to improve engagement and profitability.However, traditional segmentation approaches often fail to fully capture complex and non-linear behavioral patterns in large-scale E-commerce datasets^4,5^. In parallel, predictive models built directly on raw transactional data may suffer from noise, high dimensionality, and lack of meaningful structure. This creates a gap between customer segmentation and predictive modeling, where both tasks are often treated independently rather than as a unified framework.Recent advancements in machine learning and deep learning have significantly improved the ability to model customer behavior. In particular, Long Short-Term Memory (LSTM) and Gated Recurrent Unit (GRU) networks have demonstrated strong capability in learning sequential dependencies within transactional data^6^. These models are capable of capturing temporal dynamics and non-linear relationships that traditional machine learning models such as Logistic Regression and Support Vector Machines fail to represent effectively^7^.Despite these advances, most existing studies either focus on clustering techniques without predictive modeling or apply deep learning models without leveraging structured customer segmentation. This lack of integration limits the interpretability and effectiveness of churn prediction systems in real-world applications.Unlike conventional approaches, deep learning-based frameworks provide the ability to model dynamic customer behavior and identify high-risk customers with improved accuracy. These methods have been successfully applied in recommendation systems, fraud detection, and customer analytics, demonstrating their flexibility and robustness in handling complex datasets.

### Research gap

Despite significant progress in churn prediction and customer segmentation, several limitations remain in the existing literature. First, most studies treat customer segmentation and churn prediction as separate processes, leading to a lack of unified analytical frameworks. Second, traditional machine learning models rely heavily on handcrafted features and fail to capture deep behavioral representations. Third, many deep learning approaches ignore the importance of structured segmentation, which can enhance predictive performance and interpretability.These limitations motivate the development of a unified framework that integrates representation learning, deep clustering, and sequential modeling for more accurate and interpretable churn prediction.

### Novelty of the study

To address these limitations, this study proposes a unified hybrid framework that integrates RFM-based feature engineering, Deep Embedded Clustering (DEC), and sequential deep learning models (LSTM and GRU).The proposed framework leverages DEC to enhance feature representation by learning compact latent embeddings, which improve cluster separability and facilitate more effective predictive modeling.Unlike conventional approaches, the proposed method enables a unified workflow for customer segmentation and churn prediction through improved feature representation, rather than treating them as independent tasks.This integration allows for more robust modeling of customer behavior across different data characteristics.

### Contributions

The main contributions of this study are summarized as follows:

- **Unified Hybrid Framework:** A novel framework combining RFM analysis, Deep Embedded Clustering (DEC), and deep learning models for integrated customer segmentation and churn prediction.
- **Enhanced Feature Representation:** The use of DEC to transform customer data into meaningful latent representations, improving cluster quality and predictive performance.
- **Robust Cross-Dataset Evaluation:** Validation on two heterogeneous datasets (Online Retail and Events), demonstrating strong generalization across structured and complex data.
- **Comprehensive Model Comparison:** Evaluation of both traditional (Logistic Regression, SVM) and deep learning models (LSTM, GRU), providing insights into model behavior under different data conditions.
- **Statistical Validation:** Performance is assessed using multiple runs and statistical significance testing to ensure robustness and reliability.

### Research objectives

The main objective of this study is to develop and evaluate a hybrid framework for customer churn prediction by integrating RFM features with Deep Embedded Clustering (DEC) and deep learning models. To ensure measurable and testable objectives, the study aims to:

- Evaluate the performance of the proposed framework on the Online Retail and Events datasets using standard metrics, including accuracy, precision, recall, F1-score, and ROC-AUC.
- Compare the proposed approach against baseline models, including Logistic Regression,and Support Vector Machine (SVM).
- Analyze the impact of DEC-based feature representation on model performance by comparing results with and without clustering-based embeddings.
- Investigate the robustness of the proposed framework under different churn definitions by conducting threshold sensitivity analysis (e.g., 30, 60, and 120 days).
- Evaluate class-wise performance to ensure balanced prediction across churn and non-churn classes.

## Background and literature review

### Customer segmentation

Customer segmentation is a fundamental technique in customer analytics that involves dividing customers into distinct groups based on shared characteristics such as purchasing behavior, preferences, and transaction patterns^8,9^.By grouping customers into homogeneous segments, organizations can better understand customer needs and design targeted marketing strategies.Several segmentation approaches have been proposed in the literature, including demographic, psychographic, and behavioral segmentation^10,11^. Among these, behavioral segmentation is particularly relevant in e-commerce, as it focuses on actual customer interactions and transaction history.

### RFM analysis

Recency, Frequency, and Monetary (RFM) analysis is one of the most widely used behavioral segmentation techniques^12^. It evaluates customer value based on three key dimensions^13,14^:

- **Recency (R):** Measures how recently a customer made a purchase.
- **Frequency (F):** Represents how often a customer makes transactions.
- **Monetary (M):** Indicates the total amount spent by the customer.

RFM analysis has been extensively used in customer relationship management to identify high-value customers and predict future behavior. However, it is limited by its reliance on handcrafted features and inability to capture complex patterns in large datasets.

### Clustering techniques in customer segmentation

Clustering techniques are commonly used to automatically group customers without predefined labels^15^.Traditional clustering methods such as K-means have been widely applied due to their simplicity and efficiency^10,16^. However,these methods rely on distance-based metrics and often fail to capture non-linear relationships in high-dimensional data.Recent studies have explored advanced clustering techniques that incorporate representation learning to improve clustering quality. These approaches aim to learn more meaningful feature spaces before performing clustering.

### Deep embedded clustering (DEC)

Deep Embedded Clustering (DEC) is a deep learning-based clustering approach that combines representation learning with clustering in a unified framework^17,18^. It utilizes an autoencoder to learn a low-dimensional latent space, followed by an iterative clustering process that refines cluster assignments^19^.Compared to traditional clustering methods, DEC has demonstrated comparable performance in handling complex and high-dimensional data. It allows for better separation of clusters and improved interpretability of learned representations. However, its application in customer analytics, particularly in churn prediction, remains relatively limited.Most existing studies focus on traditional clustering methods or apply deep learning models independently, without leveraging DEC-based segmentation within a unified predictive framework.

### Churn prediction using machine learning

Customer churn prediction has been widely studied using traditional machine learning techniques such as Logistic Regression (LR), Support Vector Machines (SVM), and Random Forest^20,21^.These models are effective in structured datasets but often struggle with capturing complex behavioral patterns and temporal dependencies.Moreover, traditional models require extensive feature engineering and may not generalize well across different datasets with varying characteristics^22,23^.

### Deep learning for customer behavior modeling

Deep learning approaches have gained significant attention in recent years due to their ability to model complex and non-linear relationships. In particular:

- Long Short-Term Memory(LSTM) networks are widely used for modeling sequential and time-dependent data^6^.
- Gated Recurrent Unit (GRU) networks offer a simplified architecture with comparable performance and reduced computational cost^24^.

These models are capable of capturing temporal patterns in customer transactions, making them highly suitable for churn prediction tasks^25^.

### Summary and research direction

Based on the reviewed literature, it is evident that there is a need for an integrated approach that combines customer segmentation and churn prediction within a unified framework. This study addresses this gap by proposing a hybrid model that leverages RFM features, Deep Embedded Clustering, and deep learning models (LSTM and GRU) to improve both segmentation quality and predictive performance.

## Proposed framework

**Figure 1:**
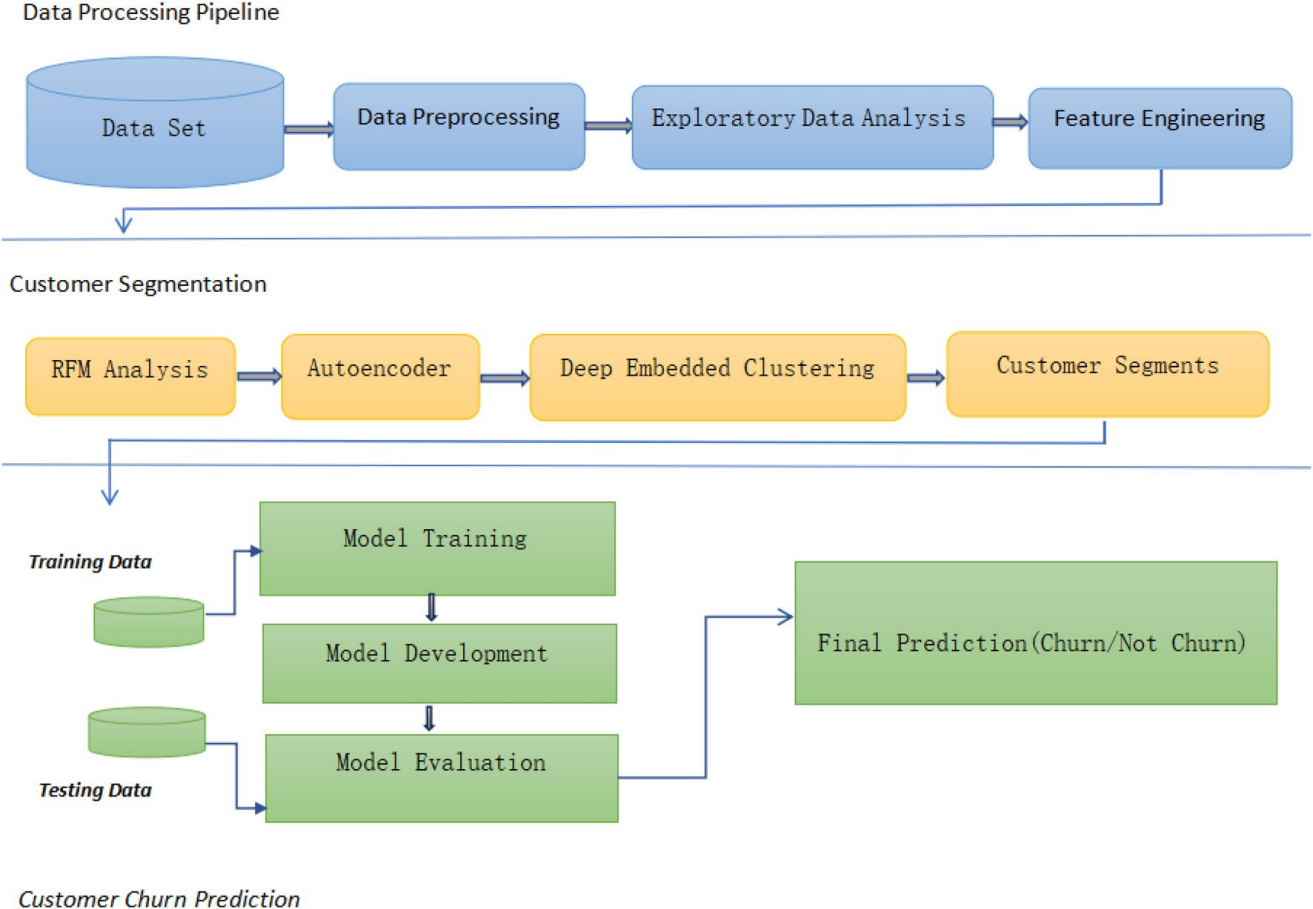
Step-by-step workflow of the proposed churn prediction pipeline. The process begins with data preprocessing and RFM feature extraction, followed by representation learning using an autoencoder and DEC clustering. The resulting features are fused and used to train multiple classification models, which are evaluated using standard performance metrics to generate final churn predictions.

As illustrated in Fig. 1,the proposed methodology introduces a hybrid framework for customer churn prediction in E-commerce by integrating Recency, Frequency, and Monetary (RFM) analysis, Deep Embedded Clustering (DEC), and deep learning models, including Long Short-Term Memory (LSTM) and Gated Recurrent Unit (GRU).The framework aims to address the challenges of customer segmentation and churn prediction by combining behavioral analytics with advanced representation learning techniques. The process begins with preprocessing transactional data, where RFM features are computed for each customer to capture purchasing behavior.These features are then provided as input to the DEC model, which learns latent representations and generates meaningful customer segments in an embedded feature space. This step enhances the separability of customer groups and captures complex behavioral patterns. Customers belonging to low-value clusters, characterized by low purchase frequency, low monetary value, and prolonged inactivity, are labeled as churn, while others are considered active.Subsequently, LSTM and GRU models are trained on the transformed data to predict customer churn. Model performance is evaluated against traditional machine learning approaches, including Logistic Regression, and Support Vector Machine, using standard evaluation metrics such as accuracy, precision, recall, F1-score, and ROC-AUC.The integration of RFM analysis, DEC, and deep learning provides a unified framework that enhances feature representation and improves predictive performance. This approach enables businesses to identify at-risk customers and design effective retention strategies.

### Dataset

The primary dataset employed in this study is the Online Retail dataset, which contains transactional records from a UK-based E-commerce platform (Online Retail – UCI Machine Learning Repository)^26^. Additionally, an Events dataset was utilized to evaluate the proposed framework in a different context, capturing user interactions and behavioral patterns relevant to customer engagement and churn^27^.

### Data preprocessing

Data preprocessing is a critical step in customer segmentation and churn prediction , particularly in deep learning applications where model performance is highly sensitive to data quality and feature distributions^28,29^. In this study, preprocessing is performed separately for the Online Retail dataset and the Events dataset to address their distinct structural characteristics.

**The Online Retail dataset** consists of transactional records representing customer purchases. Several preprocessing steps were applied to ensure data quality and consistency.First, duplicate transactions were removed to eliminate redundancy. Records with invalid values, such as negative quantities or zero prices, were excluded, as they typically correspond to canceled or erroneous transactions. Formally, let

1 $$\begin{aligned} \mathscr {D}_r = \{(c_i, t_{ij}, q_{ij}, p_{ij})\} \end{aligned}$$

denote the raw transaction dataset, where $c_i$ represents customer *i*, $t_{ij}$ is the timestamp of transaction *j*, $q_{ij}$ is the purchased quantity, and $p_{ij}$ is the unit price. Only transactions satisfying $q_{ij} > 0$ and $p_{ij} > 0$ were retained. Following data cleaning, customer-level behavioral features were constructed using the RFM model. Recency, Frequency, and Monetary value are defined as:

2 $$\begin{aligned} R_i = T - \max _j(t_{ij}), \end{aligned}$$

3 $$\begin{aligned} F_i = \sum _j 1, \end{aligned}$$

4 $$\begin{aligned} M_i = \sum _j q_{ij} \times p_{ij}, \end{aligned}$$

where *T* denotes the end of the observation period.In addition to RFM features, statistical features such as inter-purchase time, transaction variance, and purchase regularity were extracted to capture temporal dynamics and behavioral consistency.

**The Events dataset** consists of user interaction logs (e.g., views, clicks, and purchases), which naturally form sequential behavioral data.Each event is associated with a *user_session* identifier, which represents a continuous sequence of user activity within a single visit. In this study, session boundaries are directly derived from the provided *user_session* field. This ensures that session definitions reflect the platform’s native interaction structure.For each user, events are organized into ordered sequences:

5 $$\begin{aligned} \mathbf{E}_i = \{e_{i1}, e_{i2}, \ldots , e_{iT}\}, \end{aligned}$$

where events within the same *user_session* are treated as a single behavioral sequence. Data cleaning was performed to remove missing, inconsistent, and noisy records. Additionally, categorical event attributes were encoded into numerical representations suitable for deep learning models. This approach preserves the natural temporal structure of user interactions and enables effective modeling of sequential behavior patterns.

#### Exploratory data analysis (EDA)

Exploratory Data Analysis (EDA) was employed to thoroughly examine the structure and characteristics of the data prior to building predictive models^30^. This stage involved evaluating completeness, consistency, distribution patterns, and correlations among variables to support preprocessing decisions and model selection. Quantitative, non-visual techniques including summary statistics such as mean, median, and standard deviation, along with correlation analysis were used to uncover relationships between variables . In parallel, graphical techniques such as histograms, box plots, and scatter plots facilitated the visualization of distributions, identification of outliers, and detection of behavioral trends. In this study, EDA was applied to two datasets the Events dataset and the Online Retail dataset to develop a deep understanding of customer behavior and identify patterns significant to churn prediction. The Events dataset analysis focused on user engagement behaviors, including frequency of interactions, event types, and session duration. Visual analytic revealed that a considerable number of users exhibited declining activity levels over time, signaling an elevated churn risk. Additionally, correlations between certain event types and engagement intensity were identified, offering valuable insights for feature engineering, such as “interaction frequency” and “event-based engagement scores.” The Online Retail dataset was analyzed to investigate transaction history and seasonality in customer spending. Findings indicated statistically significant fluctuations in consumer purchasing, characterized by spending surges during promotional and holiday seasons and noticeable declines during off-peak periods. These observations informed the creation of temporal features, including “holiday purchase activity” and “quarterly spending trends,” improving the model’s capacity to capture seasonal churn dynamics. The insights derived from EDA were fundamental in shaping the preprocessing and feature engineering phases and ensuring that the datasets were optimally prepared for modeling. By understanding the unique behavioral and transactional characteristics of both datasets, targeted retention strategies could be designed, thereby enhancing the performance and predictive accuracy of the churn models.

### RFM feature engineering

To effectively capture customer purchasing behavior, the RFM (Recency, Frequency, Monetary) model is employed as a fundamental feature engineering technique. RFM is widely used in customer analytics due to its simplicity, interpretability, and effectiveness in representing user engagement and value. For each customer $c_i$, three key behavioral features are computed based on transactional data:

- **Recency (***R***):** measures how recently a customer made a purchase. It is defined as the time difference between the end of the observation period *T* and the most recent transaction:
  6 $$\begin{aligned} R_i = T - \max _j (t_{ij}) \end{aligned}$$
- **Frequency (***F***):** represents the total number of transactions performed by a customer:
  7 $$\begin{aligned} F_i = \sum _j 1 \end{aligned}$$
- **Monetary (***M***):** indicates the total amount spent by the customer:
  8 $$\begin{aligned} M_i = \sum _j q_{ij} \times p_{ij} \end{aligned}$$

These features provide complementary insights into customer behavior. Recency captures customer activity level, Frequency reflects engagement intensity, and Monetary measures customer value.To enhance the descriptive power of RFM features, additional statistical attributes are derived, including inter-purchase time, transaction variance, and purchase regularity. These features help capture temporal dynamics and behavioral consistency, which are essential for identifying churn patterns.Before being used in downstream tasks, all features are normalized using z-score standardization to ensure comparable scales and improve model convergence. Overall, the RFM feature set provides a compact yet informative representation of customer behavior, serving as an effective input for both clustering and churn prediction models.

### Churn label definition

Since explicit churn labels are not available in the dataset, a rule-based approach was adopted to define customer churn. A customer is considered to have churned if no activity is observed for a period exceeding 90 days. Accordingly, a binary churn variable was constructed based on the Recency feature: the threshold of 90 days was selected based on common practices in E-commerce analytics, where prolonged inactivity is considered a strong indicator of customer disengagement.

9 $$\begin{aligned} \text {Churn} = {\left\{ \begin{array}{ll} 1, & \text {if Recency} > 90 \\ 0, & \text {otherwise} \end{array}\right. } \end{aligned}$$

It is important to clarify that the churn labels are not derived from the clustering results. Instead, clustering is used solely for customer segmentation based on behavioral patterns, while churn labels are independently defined using a recency-based threshold.Specifically, customers whose recency exceeds a predefined threshold are labeled as churned (1), indicating inactivity, whereas customers with more recent transactions are labeled as non-churn (0).The cluster assignments obtained from the DEC model are incorporated as additional features in the predictive model rather than being used to define churn labels. This separation ensures that the clustering process enhances feature representation without introducing bias into the labeling mechanism.

### Feature combination for churn prediction

To construct an effective input representation for churn prediction, multiple feature sources were integrated into a unified dataset. Specifically, Recency, Frequency, and Monetary (RFM) features were combined with cluster assignments obtained from the Deep Embedded Clustering (DEC) model, along with relevant behavioral attributes.This integration enables the model to leverage both handcrafted behavioral features and latent representations learned through deep clustering, thereby improving predictive performance and interpretability. Table 1 presents a sample of the constructed dataset, which combines RFM features, cluster labels, and churn indicators. This unified representation serves as the input for subsequent churn prediction models (Table 2).

**Table 1:** Sample of events RFM data with churn labels.

User ID	Recency	Frequency	Monetary	Cluster	Churn
1515915625353286144	148	1	119.03	3	1
1515915625353475152	152	1	55.16	0	1
1515915625353534720	145	3	57.15	3	1
1515915625353561600	95	2	345.72	2	1
1515915625353900032	136	2	57.85	3	1

**Table 2:** Sample of online retail RFM data with churn labels.

CustomerID	Recency	Frequency	Monetary	Cluster	Churn
12346	326	1	77183.60	3	1
12347	2	7	4310.00	1	0
12348	75	4	1797.24	1	0
12349	19	1	1757.55	0	0
12350	310	1	334.40	2	1

To ensure comparability across features with different scales, normalization was applied to the RFM variables. The scaled features are presented in Table 3.

**Table 3:** Scaled RFM features with cluster labels and churn (events).

User ID	Recency_Scaled	Frequency_Scaled	Monetary_Scaled	Cluster	Churn
1515915625353286144	1.075318	−0.660010	0.111449	3	1
1515915625353475152	1.104240	−0.660010	−0.441104	0	1
1515915625353534720	1.053113	1.144704	−0.415772	3	1
1515915625353561600	0.595396	0.395680	0.883150	2	1
1515915625353900032	0.983651	0.395680	−0.407067	3	1

The normalization process improves model convergence and stability, particularly for deep learning models such as LSTM and GRU, which are sensitive to feature scaling.Overall, the combined feature set provides a comprehensive representation of customer behavior by integrating statistical features (RFM) with learned cluster structures (DEC). This hybrid representation bridges the gap between unsupervised segmentation and supervised learning, enabling more accurate and robust churn prediction.

### Autoencoder architecture

The autoencoder used in the DEC framework consists of a deep fully connected encoder-decoder architecture designed to learn meaningful latent representations of RFM features.The encoder is composed of multiple dense layers with decreasing dimensionality. Specifically, the input layer of size 3 (corresponding to Recency, Frequency, and Monetary) is followed by hidden layers of sizes 64 and 32 with ReLU activation functions. The data is then mapped into a latent space of dimension 10 using a linear activation function.The decoder mirrors the encoder structure, reconstructing the original input through layers of sizes 32 and 64 with ReLU activation, followed by an output layer of size 3 with sigmoid activation to ensure normalized output values.The autoencoder is trained using the Mean Squared Error (MSE) loss function^31^ and optimized using the Adam optimizer.After training, the encoder is used to generate latent embeddings, which are then used as input to the DEC clustering algorithm, improving clustering quality and representation learning. The architecture of the autoencoder used in the DEC framework is presented in Table 4. The model adopts a symmetric encoder-decoder structure to ensure effective reconstruction of input features. The encoder progressively reduces the dimensionality of the input RFM features from 3 to a latent representation of size 10, enabling the model to capture underlying nonlinear patterns in customer behavior.The use of ReLU activation functions in the hidden layers enhances nonlinearity, while the linear activation in the latent layer preserves feature continuity for clustering. The decoder mirrors the encoder structure to reconstruct the original input, with a sigmoid activation function applied at the output layer to match the normalized input range.This architecture allows the model to learn compact and informative representations, which significantly improves clustering performance in the subsequent DEC stage.

**Table 4:** Autoencoder architecture used in DEC pretraining.

Layer	Type	Units	Activation
Input Layer	Dense	3 (RFM)	–
Encoder Layer 1	Dense	64	ReLU
Encoder Layer 2	Dense	32	ReLU
Latent Layer	Dense	10	Linear
Decoder Layer 1	Dense	32	ReLU
Decoder Layer 2	Dense	64	ReLU
Output Layer	Dense	3	Sigmoid

### Cluster number selection

**Figure 2:**
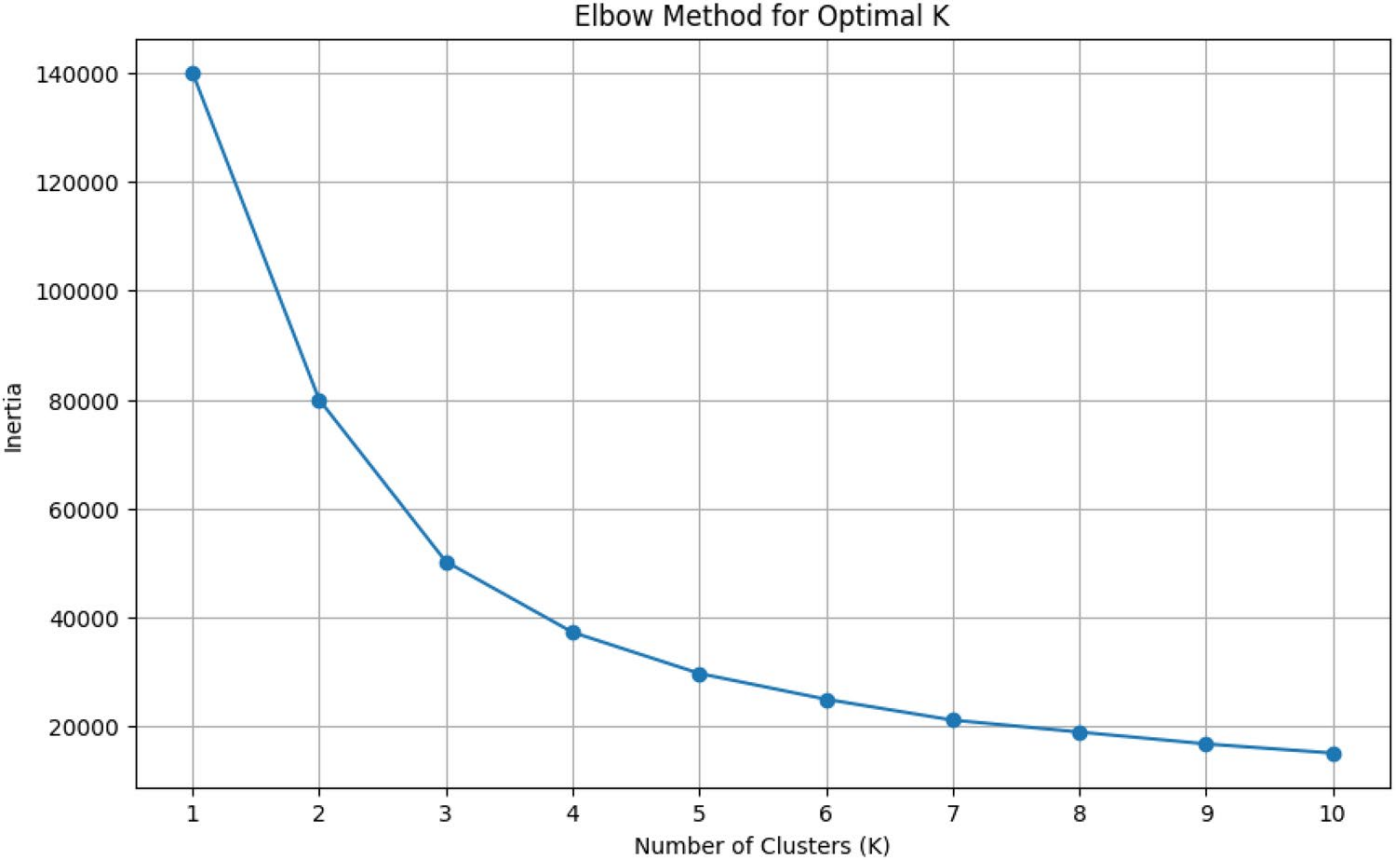
Elbow method for determining the optimal number of cluster (K).

The optimal number of clusters (K) was determined using the Elbow method. As shown in Fig. 2, the inertia decreases sharply as K increases from 1 to 3, after which the rate of decrease slows down significantly.This indicates that the optimal number of clusters lies around K=3 or K=4. Based on this observation, K=4 was selected to ensure better cluster interpretability and representation of customer segments.This choice is further supported by the t-SNE visualization Fig. 7, which shows well-separated and meaningful clusters when K=4 is used.

### Deep embedded clustering (DEC)

Deep Embedded Clustering (DEC) is employed to learn compact and discriminative latent representations of customer behavior while simultaneously performing clustering in the embedded space. The process in algorithm1 begins with a pretraining phase, where an autoencoder is trained using Mean Squared Error (MSE) loss and the Adam optimizer to reconstruct the input data and capture its underlying structure. The encoder is then used to transform the original feature matrix *X* into a lower-dimensional latent representation $Z = f_{\theta }(X)$.To initialize clustering, the *k*-means algorithm is applied to the latent space *Z* to obtain initial cluster centroids. DEC then iteratively refines cluster assignments using a soft assignment mechanism based on Student’s t-distribution, where the similarity between each embedded point $z_i$ and centroid $\mu _k$ is computed to produce soft labels $q_{ik}$.A target distribution $p_{ik}$ is subsequently derived from $q_{ik}$ to emphasize high-confidence assignments and improve cluster purity. The model is optimized by minimizing the Kullback–Leibler (KL) divergence between the target distribution and the soft assignments. During each iteration, both the encoder parameters and cluster centroids are updated via backpropagation using the Adam optimizer.The optimization process continues until convergence, defined by a small change in cluster assignments below a predefined threshold $\epsilon = 10^{-8}$. Finally, each data point is assigned to the cluster with the highest probability, resulting in refined and semantically meaningful customer segments that enhance downstream churn prediction performance.

**Algorithm 1:**
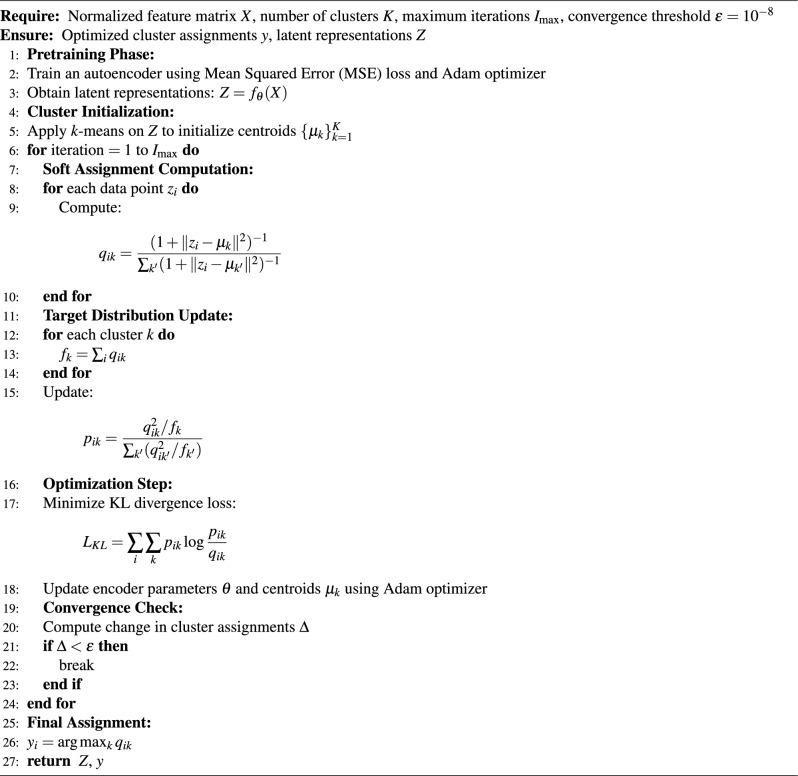
Deep Embedded Clustering (DEC) Optimization with KL-Divergence Loss

## Apply classification algorithms

To evaluate the effectiveness of the proposed framework, both deep learning and traditional machine learning models were applied, including Long Short-Term Memory (LSTM), Gated Recurrent Unit (GRU), Support Vector Machine (SVM), and Logistic Regression (LR).Deep learning models, particularly LSTM and GRU, are well-suited for capturing temporal dependencies in sequential customer behavior data. The LSTM model was selected for its strong capability in modeling long-term dependencies, while the GRU was adopted as a computationally efficient alternative with comparable performance.In addition, traditional machine learning models, were implemented as baseline methods to provide a comparative evaluation and highlight the advantages of deep learning approaches.To ensure a fair comparison, all models were trained and evaluated using the same dataset, preprocessing steps, and experimental settings.

### Architectures of deep learning models

Using the derived customer segments and RFM features, the deep learning models were implemented using a sequential architecture in Keras^32^. Table 5 summarizes the architecture and hyperparameters of the proposed LSTM and GRU models, including recurrent layers, activation functions, and training configurations.The models were trained using the Adam optimizer with a learning rate of 0.001 and binary cross-entropy as the loss function^33^.Training was conducted for 50 epochs with a batch size of 32 and a validation split of 20% to monitor generalization performance. These configurations allow the models to effectively learn meaningful patterns from sequential data while maintaining computational efficiency.

To capture temporal dependencies in customer behavior, a Long Short-Term Memory (LSTM) network was employed as part of the proposed framework.The model consists of two stacked LSTM layers with 64 and 32 hidden units, respectively, followed by fully connected dense layers. The first LSTM layer extracts sequential patterns from the input data, while the second layer refines the learned representation.The output from the LSTM layers is passed to a dense layer with 16 neurons for feature transformation, followed by a final dense layer with a sigmoid activation function to produce binary churn predictions.

In addition to the LSTM model, a Gated Recurrent Unit (GRU) network was employed to model sequential customer behavior with a more computationally efficient architecture.The GRU model consists of two stacked GRU layers with 64 and 32 hidden units, respectively. These layers are designed to capture temporal dependencies while reducing model complexity compared to LSTM.The extracted features are then passed to a fully connected dense layer with 16 neurons, followed by a final dense layer with a sigmoid activation function for binary churn prediction.Both LSTM and GRU architectures were designed with similar structures to ensure a fair comparison between models.

**Table 5:** Hyperparameters and architectures of LSTM and GRU models.

Component	GRU model	LSTM model
Recurrent layers	Layer 1: GRU (64 units, tanh) Layer 2: GRU (32 units, tanh)	Layer 1: LSTM (64 units, tanh) Layer 2: LSTM (32 units, tanh)
Dropout	0.2	0.2
Dense layers	Dense (16 units, ReLU)	Dense (16 units, ReLU)
Output layer	Sigmoid (1 unit)	Sigmoid (1 unit)
Optimizer	Adam (learning rate = 0.001)	Adam (learning rate = 0.001)
Loss function	Binary cross-entropy	Binary cross-entropy
Batch size	32	32
Epochs	50	50
Validation split	20% of training data	20% of training data
Random seed	42 (for reproducibility)	42 (for reproducibility)

### Data splitting

The dataset was divided into training and testing sets using an 80/20 split. Specifically, 80% of the data was used for training the models, while the remaining 20% was reserved for testing.To ensure a fair evaluation, the split was performed in a stratified manner based on the churn label, preserving the class distribution in both training and testing sets.Additionally, 20% of the training data was used as a validation set during model training to monitor performance and prevent overfitting.

### Optimization

Model parameters were optimized using the Adam optimizer, which combines the advantages of momentum-based gradient descent and adaptive learning rate methods^34,35^. Let $\theta _t$ denote the model parameters at iteration *t*, and let $g_t = \nabla _{\theta } \mathcal {L}(\theta _t)$ be the gradient of the loss function $\mathcal {L}$ with respect to $\theta _t$. Adam maintains exponentially decaying estimates of the first-order moment (mean) and second-order moment (uncentered variance) of the gradients, given by

10 $$\begin{aligned} m_t = \beta _1 m_{t-1} + (1 - \beta _1) g_t, \end{aligned}$$

11 $$\begin{aligned} v_t = \beta _2 v_{t-1} + (1 - \beta _2) g_t^2, \end{aligned}$$

where $\beta _1$ and $\beta _2$ are the decay rates controlling the momentum terms.

To counteract the bias introduced by initialization at zero, bias-corrected estimates are computed as

12 $$\begin{aligned} \hat{m}_t = \frac{m_t}{1 - \beta _1^t}, \quad \hat{v}_t = \frac{v_t}{1 - \beta _2^t}. \end{aligned}$$

The parameter update rule is then defined as

13 $$\begin{aligned} \theta _{t+1} = \theta _t - \alpha \frac{\hat{m}_t}{\sqrt{\hat{v}_t} + \epsilon }, \end{aligned}$$

where $\alpha$ denotes the learning rate and $\epsilon$ is a small constant added for numerical stability. In this study, the learning rate was fixed at $\alpha = 0.001$, while the default values $\beta _1 = 0.9$, $\beta _2 = 0.999$, and $\epsilon = 10^{-8}$ were used.

The adaptive scaling of parameter-wise learning rates enables Adam to achieve fast convergence and stable optimization, making it particularly well-suited for training recurrent neural networks such as LSTM and GRU models. To ensure experimental consistency, the same optimization configuration was applied across all evaluated architectures. Algorithm 2 summarizes the optimization procedure used in this study.

**Algorithm 2:**
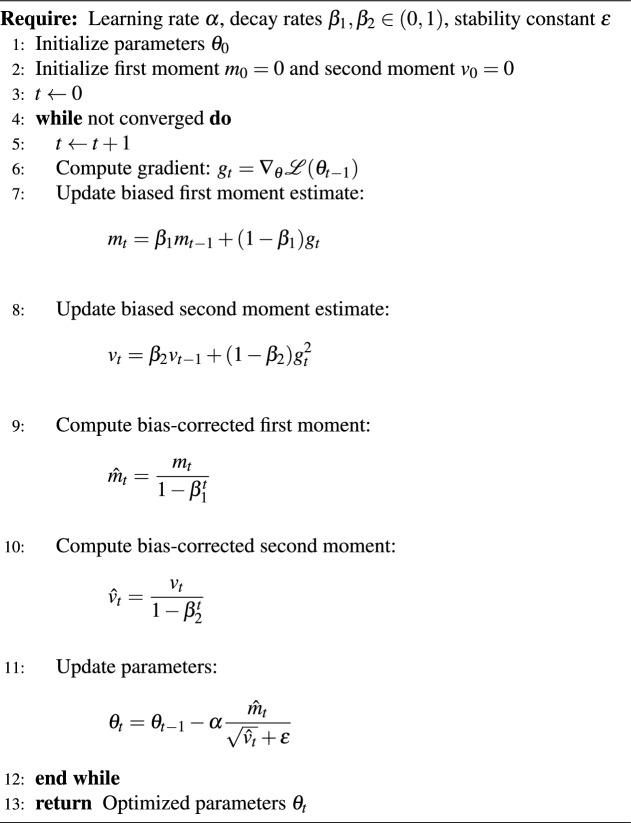
Adam Optimization Algorithm

### Evaluation metrics

The performance of the proposed models was evaluated using standard classification metrics derived from the confusion matrix, including accuracy, precision, recall, F1-score, and the area under the receiver operating characteristic curve (AUC–ROC). These metrics provide a comprehensive assessment of model effectiveness by capturing both overall predictive performance and the trade-off between false positives and false negatives^36^.

Let *TP*, *TN*, *FP*, and *FN* denote the number of true positives, true negatives, false positives, and false negatives, respectively. The evaluation metrics are defined as follows:

14 $$\begin{aligned} \text {Accuracy} = \frac{TP + TN}{TP + TN + FP + FN} \end{aligned}$$

15 $$\begin{aligned} \text {Precision} = \frac{TP}{TP + FP}, \quad \text {Recall} = \frac{TP}{TP + FN} \end{aligned}$$

16 $$\begin{aligned} F1 = \frac{2 \cdot \text {Precision} \cdot \text {Recall}}{\text {Precision} + \text {Recall}} \end{aligned}$$

In addition, the AUC–ROC metric was employed to evaluate the discriminative capability of the models across different classification thresholds.To ensure a fair and consistent comparison, all models were evaluated on the same test set using identical preprocessing steps and training configurations.These metrics were selected to ensure a balanced evaluation, particularly in the presence of class imbalance.

### Implementation details

All experiments were implemented using Python 3.10. Deep learning models were developed using TensorFlow with the Keras API, while traditional machine learning models were implemented using scikit-learn.The autoencoder was trained using the Adam optimizer with a learning rate of 0.001 and Mean Squared Error (MSE) as the reconstruction loss function. The learned latent representations were then used as input for the Deep Embedded Clustering (DEC) algorithm. For the sequential models, both LSTM and GRU architectures were trained using binary cross-entropy as the loss function and the Adam optimizer. The models were trained for 50 epochs with a batch size of 32, and a validation split of 20% was used to monitor training performance.To ensure reproducibility, all experiments were conducted using a fixed random seed (42).The experiments were performed on a machine equipped with an Intel Core i5-8400H CPU @ 2.50 GHz, 16 GB RAM, and a GPU with 4 GB memory. This setup ensures reproducibility while maintaining a balance between computational efficiency and model performance. No specialized high-performance computing resources were required.

### Computational performance analysis

To provide a comprehensive evaluation of model efficiency, both computational complexity and runtime performance were analyzed, including the number of parameters, memory usage, prediction latency, and training time. The results show that deep learning models exhibit higher complexity compared to traditional methods. Specifically, the LSTM model contains 30,625 parameters and requires approximately 0.117 MB of memory, while the GRU model has fewer parameters (23,393) and lower memory usage (0.089 MB), reflecting its more compact architecture. In terms of runtime performance, GRU achieves slightly lower prediction latency (86.75 ms) compared to LSTM (92.11 ms), indicating more efficient inference. However, both models require significantly higher training time compared to traditional machine learning models due to their sequential nature and increased representational capacity. Overall, these results highlight a trade-off between model complexity and computational efficiency. While deep learning models provide more expressive representations, GRU offers a more efficient alternative with reduced computational cost and comparable predictive performance.

## Experiments and results

### Data description

**Online Retail Dataset** (UCI Machine Learning Repository) is a real transactional dataset that records all purchases made by a UK-based, registered non-store online retail company between December 1, 2010 and December 9, 2011. It contains 541,909 instances and several key attributes such as **InvoiceNo** (transaction identifier), **StockCode** (product code), **Description** (product name), **Quantity** (units sold), **InvoiceDate** (date and time of transaction), **UnitPrice** (price per unit), **CustomerID** (customer identifier), and **Country** (customer country). Each row represents a line-item on an invoice, enabling detailed analysis of customer purchases, product demand, and seasonal trends. This dataset is widely used for clustering, classification, and time-series modelling in retail analytics and customer segmentation tasks^26^.

**Open CDP Events Dataset**(E-commerce Behavior Data) consists of rich E-commerce user behavior records collected through a customer data platform (CDP) that logs interactions from online retail sites. Publicly available subsets include behavioral event data for various retail categories (e.g., electronics, cosmetics, multi-category stores) spanning several months, each dataset includes user behavioral events such as product **views**, **adds to cart**, **removals**, and **purchases** tied to **timestamps**, **user IDs**, **product IDs**, and other attributes like **category**, **brand**, and **price**. These event logs represent the sequence of user interactions during shopping sessions and are invaluable for analyzing browsing patterns, conversion funnels, recommendation systems, and session-based modeling^27^.

### Online retail results

#### Data analysis and statistical validation

To better understand customer behavior in the Online Retail dataset, a comprehensive statistical analysis was conducted on the RFM features (Recency, Frequency, and Monetary).

**Table 6:** Correlation matrix between RFM features and churn.

	Recency	Frequency	Monetary	Churn
Recency	1.00	−0.26	−0.12	0.86
Frequency	−0.26	1.00	0.55	−0.22
Monetary	−0.12	0.55	1.00	−0.11
Churn	0.86	−0.22	−0.11	1.00

Table 6 presents the correlation matrix between RFM features and churn. A strong positive correlation is observed between Recency and churn (approximately 0.86), indicating that customers with longer inactivity periods are more likely to churn. In contrast, Frequency and Monetary show negative correlations with churn, suggesting that highly active and high-spending customers are less likely to leave.

#### DEC clustering results

**Figure 3:**
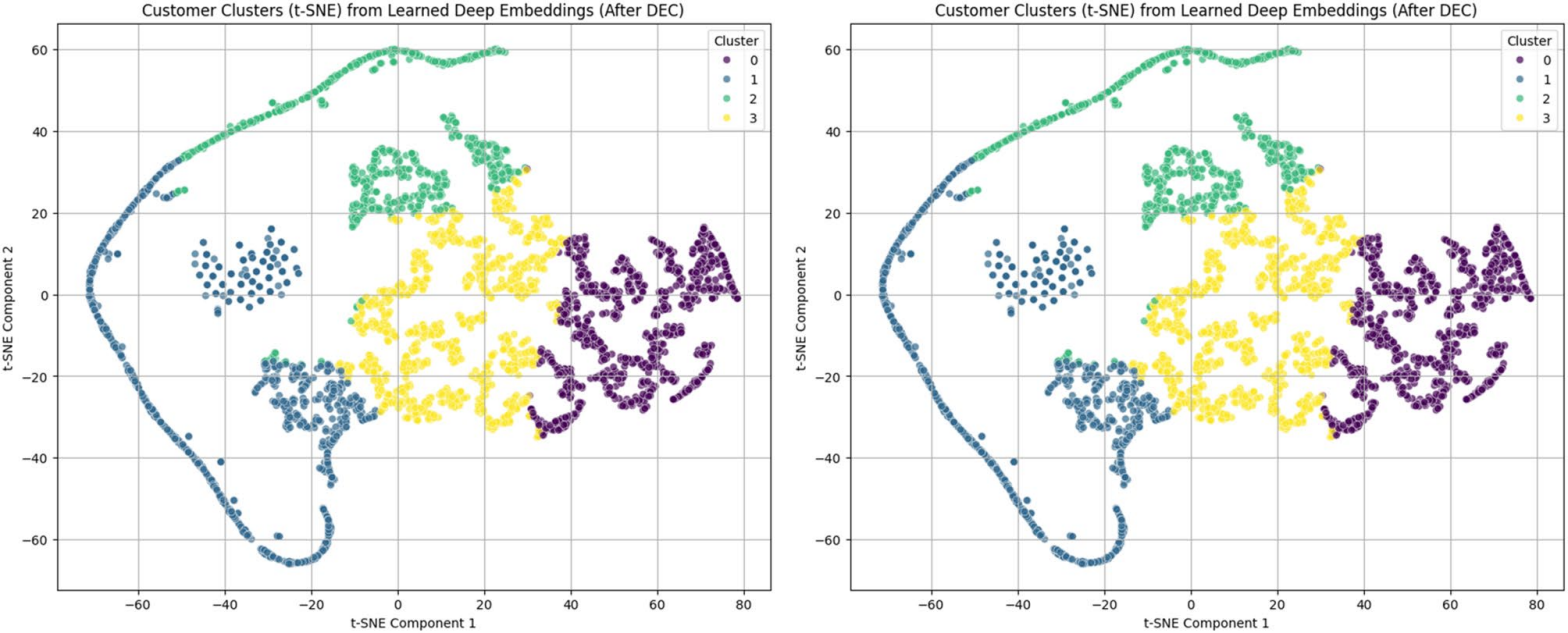
t-SNE visualizations of customer segments (**a**) before applying DEC and (**b**) after applying DEC.

To evaluate the effectiveness of the proposed clustering approach, t-SNE visualizations were employed to analyze the structure of customer segments before and after applying the Deep Embedded Clustering (DEC) model.Before applying DEC (Fig. 3), the clusters exhibit noticeable overlap and less distinct boundaries, indicating limited separability in the original RFM feature space. Although some grouping patterns can be observed, the clusters are not clearly defined, which may negatively affect downstream prediction tasks.After applying DEC (Fig. 3), a significant improvement in cluster structure is observed. The clusters become more compact, well-separated, and clearly distinguishable, with minimal overlap between groups. This demonstrates that the DEC model effectively learns a more discriminative latent representation of customer behavior.The improvement in clustering quality can be attributed to the joint optimization of feature representation and cluster assignment through the KL divergence objective in DEC. This enables the model to refine the latent space and enhance the separation between different customer groups.

**Figure 4:**
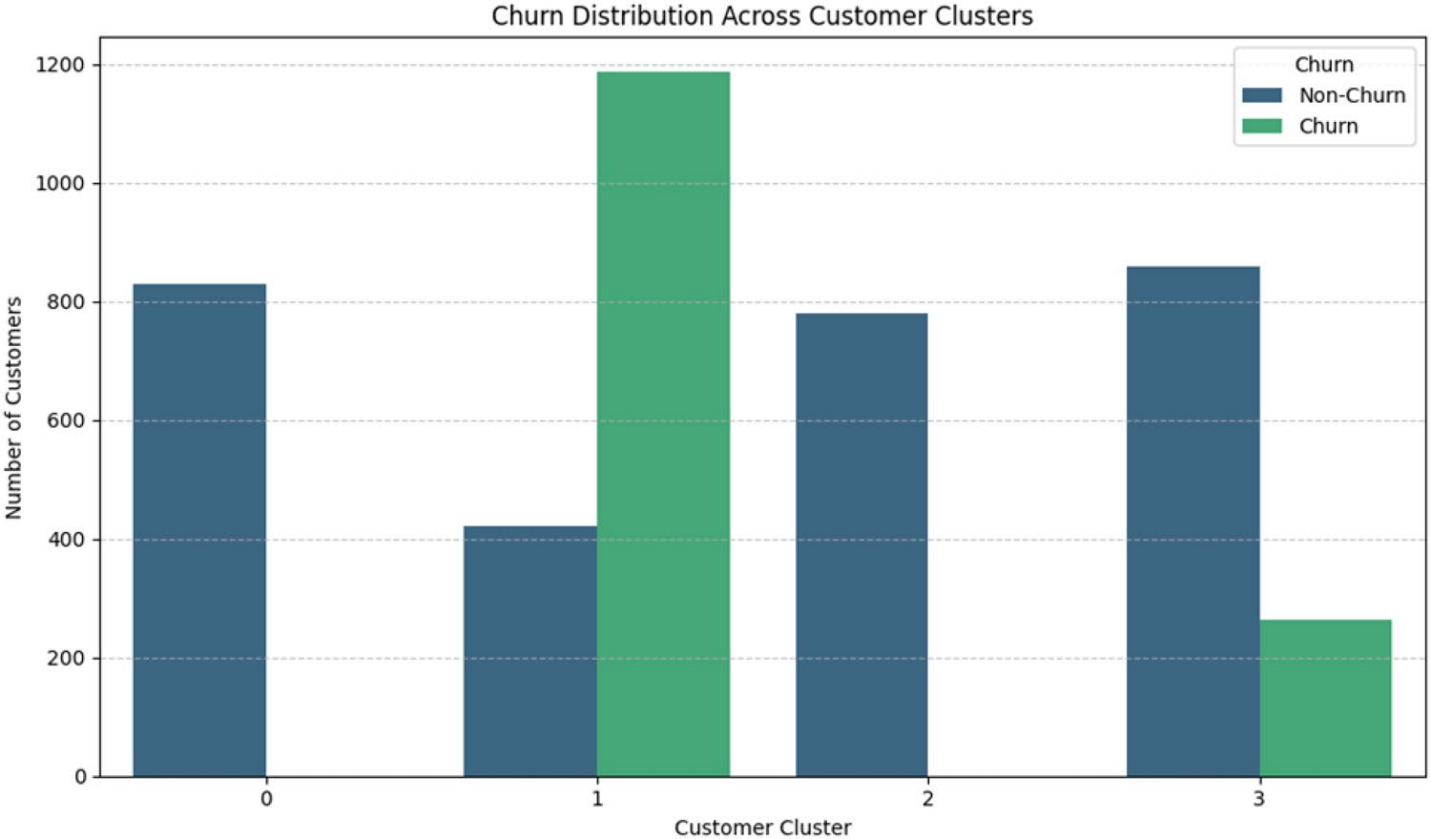
Distribution of churn and non-churn customers across clusters generated using the DEC model.

The clear separation between these clusters highlights the effectiveness of the proposed DEC-based segmentation approach. The refined customer representation provides a strong foundation for subsequent churn prediction models, as it captures underlying behavioral patterns more effectively than the original feature space. Table 7 and Fig. 4 presents a comprehensive analysis of customer distribution and churn rates across the identified clusters.The results reveal significant variation in churn behavior among the clusters. Cluster 1 exhibits the highest churn rate (73.80%), with 1186 churned customers out of 1607, clearly identifying it as a high-risk segment. This cluster represents disengaged customers with a strong tendency to churn.In contrast, Clusters 0 and 2 show zero churn rates, indicating fully stable and loyal customer groups. The absence of churn in these clusters suggests strong engagement and consistent purchasing behavior.Cluster 3 demonstrates a moderate churn rate (23.44%), representing a transitional segment. Customers in this group are partially engaged and may become churners if no intervention strategies are applied.

**Table 7:** Customer distribution and churn rates across clusters.

Cluster	Non-churn	Churn	Total	Non-churn (%)	Churn (%)
Cluster 0	830	0	830	100.00	0.00
Cluster 1	421	1186	1607	26.20	73.80
Cluster 2	779	0	779	100.00	0.00
Cluster 3	859	263	1122	76.56	23.44

Overall, the clear separation of clusters based on churn rates highlights the effectiveness of the DEC-based segmentation in capturing meaningful behavioral patterns. This segmentation enhances model interpretability and supports more targeted customer retention strategies.

#### Model performance

To evaluate the effectiveness of the proposed framework, multiple machine learning and deep learning models were employed for churn prediction, including Logistic Regression (LR), Support Vector Machine (SVM), Long Short-Term Memory (LSTM), and Gated Recurrent Unit (GRU).

**Figure 5:**
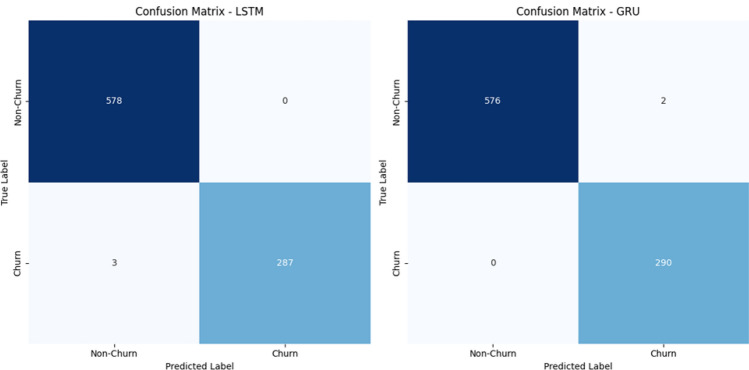
Confusion matrices for (**a**) LSTM and (**b**) GRU models on the Online Retail dataset.

The confusion matrix presented in Fig. 5 reveals that both LSTM and GRU achieve excellent classification performance.The LSTM model demonstrates zero false positives, indicating strong reliability in identifying non-churn customers. However, it slightly under-detects churn cases with a small number of false negatives.In contrast, the GRU model achieves perfect recall for churn detection, with zero false negatives, successfully identifying all churn cases. Although it introduces a small number of false positives, its ability to capture all churn instances makes it particularly effective for churn prediction tasks.It is important to note that the confusion matrices are computed using the test set only, which represents 20% of the total dataset after applying the train-test split.As a result, the number of samples shown in the confusion matrices is smaller than the overall dataset size. This is a standard evaluation practice used to assess model performance on unseen data and ensure unbiased results.

**Figure 6:**
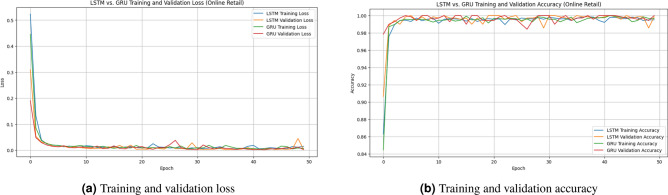
Training performance of LSTM and GRU models on the online retail dataset.

Figure 6 illustrates the training dynamics of both LSTM and GRU models in terms of loss and accuracy. Both models exhibit rapid convergence within the first few epochs, with accuracy values approaching 1.0 and loss values decreasing to near zero.The training and validation curves closely follow each other, indicating strong generalization and the absence of overfitting. The GRU model shows slightly more stable behavior with fewer fluctuations, while achieving comparable or slightly better performance than LSTM.These results confirm that the proposed framework effectively captures customer behavior patterns and enables highly accurate churn prediction.

Overall, both deep learning models achieve comparable performance, with GRU slightly outperforming LSTM due to its simpler architecture and lower computational complexity. These results validate the effectiveness of the proposed hybrid framework in accurately predicting customer churn. To provide a comprehensive evaluation, the performance of traditional machine learning models and deep learning models was compared using standard classification metrics (Table 8).The LSTM model achieves an accuracy of 0.9965 and an F1-score of 0.9948, while the GRU model slightly outperforms LSTM with an accuracy of 0.9977 and an F1-score of 0.9966. Notably, GRU achieves perfect recall (1.000), indicating its ability to correctly identify all churn cases, which is particularly important in churn prediction tasks.Traditional machine learning models also perform competitively. Logistic Regression achieves perfect precision (1.000), indicating no false positives, while SVM shows slightly lower performance compared to other models.

**Table 8:** Performance comparison on online retail dataset.

Model	Accuracy	Precision	Recall	F1-score	ROC-AUC
Logistic regression	0.9931	1.0000	0.9793	0.9895	0.99996
SVM	0.9885	0.9965	0.9690	0.9825	0.99974
LSTM	0.9965	1.0000	0.9897	0.9948	0.99993
GRU	0.9977	0.9932	1.0000	0.9966	0.99999

Table 9 further presents the macro-average and weighted-average metrics. The close similarity between macro and weighted scores across all models indicates balanced performance and robustness. This suggests that the model are not biased toward the majority class and maintain consistent performance across both churn and non-churn customers as presented in Table10.

**Table 9:** Macro and weighted average metrics on online retail dataset.

Model	F1 (macro)	F1 (weighted)	Support
Logistic regression	0.9922	0.9931	868
SVM	0.9922	0.9931	868
LSTM	1.0000	0.9965	868
GRU	0.9974	0.9977	868

**Table 10:** Class-wise performance metrics for all models.

Model	Class	Precision	Recall	F1-score	Support
Logistic regression	Non-churn (0)	0.9897	1.0000	0.9948	578
	Churn (1)	1.0000	0.9793	0.9895	290
SVM	Non-churn (0)	0.9897	1.0000	0.9948	578
	Churn (1)	0.9965	0.9793	0.9895	290
LSTM	Non-churn (0)	1.0000	0.9897	1.0000	578
	Churn (1)	1.0000	0.9965	1.0000	290
GRU	Non-churn (0)	0.9983	1.0000	0.9983	578
	Churn (1)	0.9966	1.0000	0.9966	290

Overall, The results show that all models achieve very high predictive performance, with accuracy values exceeding 0.98 across all approaches.It is important to note that although the original dataset contains over 540,000 transaction records, the analysis is performed at the customer level after applying RFM feature engineering. This aggregation transforms the dataset into a smaller set of unique customers.As a result, the evaluation is conducted on a test set of 868 customers (20% of the dataset).The results demonstrate that the proposed framework achieves highly accurate and balanced performance, with GRU providing the best trade-off between churn detection capability and generalization.

#### Impact of deep embedded clustering (DEC) on model performance

To evaluate the contribution of the clustering component, an ablation study was conducted by comparing model performance with and without the inclusion of Deep Embedded Clustering (DEC). Table 11 presents a comparative analysis of model performance on the Online Retail dataset with and without the integration of Deep Embedded Clustering (DEC),The results show that incorporating DEC significantly improves the performance of both LSTM and GRU models. Specifically, the RFM + LSTM model achieves an accuracy of 0.9928 and an F1-score of0.9912 without DEC, while the inclusion of DEC increases the accuracy to 0.9965 and the F1-score to 0.9948. Similarly, the RFM + GRU model records an accuracy of 0.9951 and an F1-score of 0.9901 without DEC. When DEC is applied, the performance improves substantially, achieving an accuracy of 0.9977 and an F1-score of 0.9966. These results demonstrate that DEC enhances feature representation by learning more discriminative latent structures, leading to improved classification performance. Overall, the proposed framework consistently outperforms the baseline models without DEC, highlighting the effectiveness of integrating clustering with deep learning for customer churn prediction.

**Table 11:** Performance comparison with and without DEC.

Configuration	Accuracy	F1-score
RFM + LSTM (without DEC)	0.9928	0.9912
RFM + GRU (without DEC)	0.9951	0.9901
RFM + DEC + LSTM (proposed)	**0.9965**	**0.9948**
RFM + DEC + GRU (proposed)	**0.9977**	**0.9966**

Significant values are in [bold].

#### Threshold analysis

To evaluate the effect of churn definition, multiple inactivity thresholds (30, 60, and 120 days) were investigated.

**Table 12:** Impact of different churn thresholds on model performance (online retail).

Threshold	Model	Accuracy	Recall	F1-score
30 days	LSTM	0.9992	1.0000	1.0000
	GRU	1.0000	1.0000	1.0000
60 days	LSTM	0.9965	1.000	0.9961
	GRU	0.9919	0.9820	0.9909
120 days	LSTM	1.0000	1.0000	1.0000
	GRU	0.9988	0.9959	1.0000

Table 12 presents the impact of different churn thresholds (30, 60, and 120 days) on model performance using the proposed DEC-based framework on the Online Retail dataset. The results indicate that both LSTM and GRU models achieve consistently high performance across all thresholds, demonstrating the robustness of the proposed approach. At a threshold of 30 days, both models achieve near-perfect results, with GRU reaching perfect accuracy, recall, and F1-score, while LSTM also achieves almost identical performance. This suggests that shorter thresholds produce a larger number of churn instances, making the classification task relatively easier.At a threshold of 60 days, a slight decrease in performance is observed, particularly for the GRU model in terms of recall and F1-score. This indicates a more balanced and realistic churn definition, where the distinction between churn and non-churn customers becomes more challenging. At a threshold of 120 days, performance improves again, with LSTM achieving perfect scores across all metrics and GRU maintaining near-perfect results. This behavior can be attributed to the stricter churn definition, where only highly inactive customers are labeled as churn, resulting in clearer class separation. The consistently strong performance across all thresholds highlights the effectiveness of integrating Deep Embedded Clustering (DEC) with RFM features. DEC enhances feature representation by learning latent structures that capture complex customer behavior patterns, leading to improved class separability regardless of the chosen churn definition.

Overall, the proposed framework demonstrates high stability and generalization capability, confirming that its performance is not sensitive to a specific churn threshold.

### Events dataset results

To evaluate the effectiveness of the proposed clustering approach on the Events dataset, t-SNE visualization was applied to the learned feature representations after Deep Embedded Clustering (DEC).

**Figure 7:**
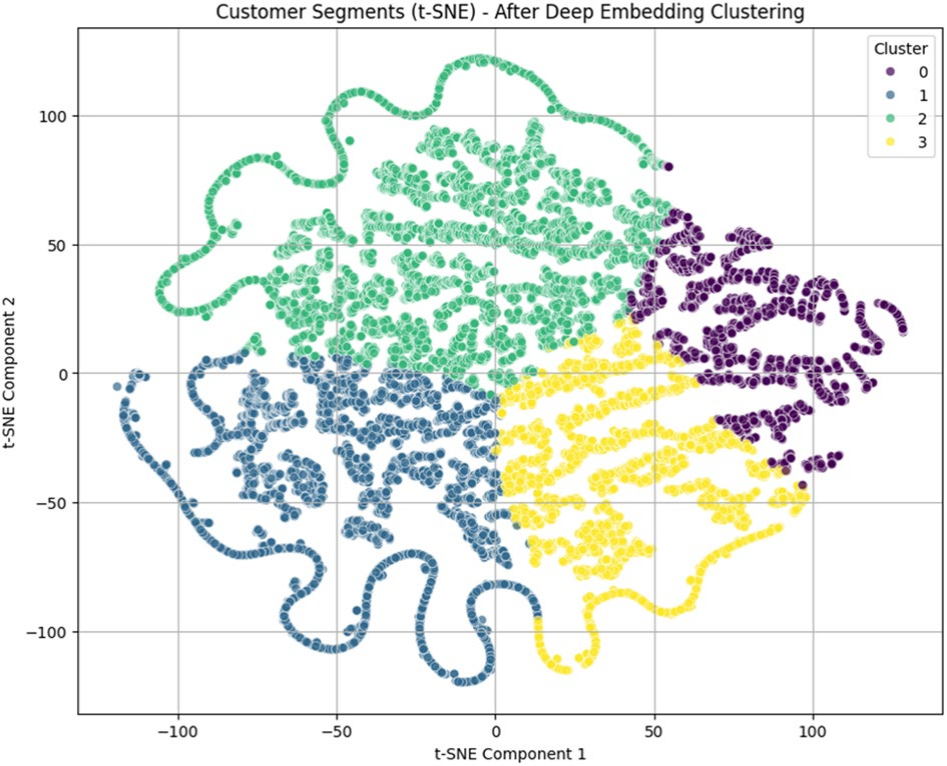
t-SNE visualization of customer segments after applying DEC on the Events dataset.

As shown in Fig. 7, the clusters are clearly separated with well-defined boundaries and minimal overlap between groups. Each cluster occupies a distinct region in the feature space, indicating strong discriminative power of the learned embeddings. Compared to raw feature representations, the DEC model significantly enhances cluster compactness and separation. This confirms that the learned latent space effectively captures underlying behavioral patterns in the Events dataset. The identified clusters correspond to meaningful customer segments, including highly active users, moderate users, low-engagement customers, and potential churn-risk groups. This structured segmentation provides a strong foundation for subsequent predictive modeling.

#### Model performance

The performance of the proposed framework was evaluated using multiple machine learning and deep learning models. The results demonstrate consistently high performance across all models.As shown in Table 13, all models achieve extremely high performance, with several models reaching perfect classification results. Deep learning models, including LSTM and GRU, demonstrate excellent performance, achieving near-perfect accuracy and ROC-AUC values close to 1.0. These results confirm the effectiveness of the proposed framework in capturing complex customer behavior patterns.

To further assess model performance across classes, both macro-average and weighted-average evaluation metrics were analyzed.The results show that the macro-average and weighted-average scores are nearly identical across all models. This indicates that the models achieve consistent performance across both churn and non-churn classes, with no significant bias toward the majority class.This behavior suggests that the dataset exhibits a relatively balanced class distribution, and that the models are capable of learning representative patterns for both classes effectively.The consistency between macro and weighted metrics further confirms the robustness and stability of the proposed approach in handling classification tasks under realistic conditions.

**Figure 8:**
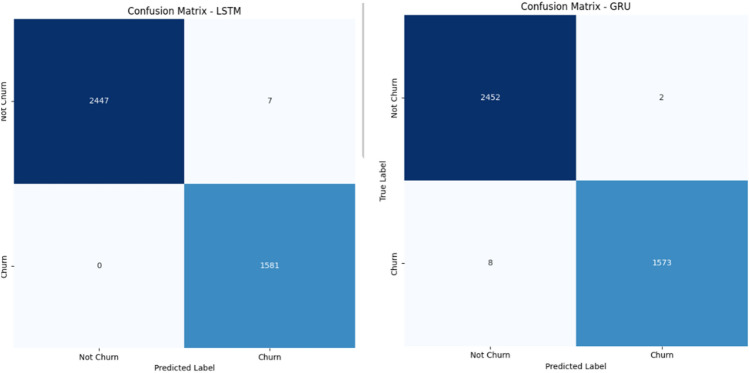
Confusion matrices for (**a**) LSTM and (**b**) GRU models on the Events dataset.

**Figure 9:**
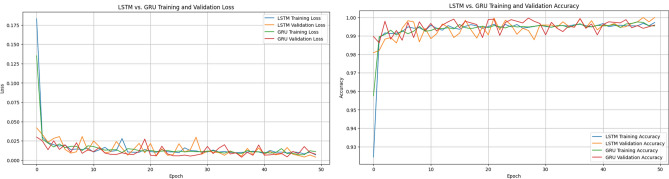
Training performance of LSTM and GRU models in terms of (**a**) loss and (**b**) accuracy.

The confusion matrices shown in Fig. 8 further confirm the high predictive performance of the models. The LSTM model correctly classifies almost all instances, with only a few misclassifications. Similarly, the GRU model demonstrates highly accurate predictions with minimal errors.The very low number of false positives and false negatives indicates strong model reliability and robustness in distinguishing churn and non-churn customers (Fig. 9).

**Table 13:** Performance comparison on the events dataset.

Model	Accuracy	Precision	Recall	F1-score	ROC-AUC
Logistic regression	0.9975	0.9937	1.0000	0.9968	1.00000
SVM	0.9975	0.9950	0.9987	0.9968	0.99998
LSTM	0.9983	0.9956	1.0000	0.9978	0.99999
GRU	0.9975	0.9987	0.9949	0.9968	0.99997

The training curves illustrate rapid convergence of both LSTM and GRU models. Loss values decrease sharply within the first few epochs and stabilize at very low levels. Similarly, accuracy quickly approaches values close to 1.0. The close alignment between training and validation curves indicates strong generalization capability and absence of overfitting. The GRU model demonstrates slightly more stable behavior, while maintaining comparable predictive performance to LSTM.

### Statistical analysis

To ensure the robustness of the proposed framework, all models were evaluated over 10 independent runs using different random seeds. The mean and standard deviation of performance metrics are reported in Table 14.All models achieve high performance across all evaluation metrics. The GRU model achieves the highest accuracy and F1-score, along with extremely low standard deviation, indicating strong stability across multiple runs. The LSTM model demonstrates slightly lower performance with higher variance, suggesting sensitivity to initialization. Traditional models such as Logistic Regression and SVM also achieve competitive results, with only minor differences between them. To further evaluate the statistical significance of the observed performance differences, pairwise Wilcoxon signed-rank tests were conducted using ROC-AUC scores^37^, as reported in Table 15. The results indicate that both LSTM and GRU significantly outperform traditional models, namely Logistic Regression and SVM (p < 0.05).However, no statistically significant difference is observed between LSTM and GRU (p> 0.05), indicating that both deep learning models achieve comparable predictive performance. Similarly,the difference between Logistic Regression and SVM is not statistically significant, confirming their comparable effectiveness.Overall,these findings suggest that the observed performance improvements are primarily driven by enhanced feature representation rather than differences in model architecture.

**Table 14:** Mean and standard deviation of model performance over 10 runs (online retail dataset).

Model	Accuracy	Precision	Recall	F1-score
Logistic Regression	0.996024 ± 0.000851	0.999933 ± 0.000210	0.993529 ± 0.001925	0.996614 ± 0.000725
SVM	0.994680 ± 0.001601	0.998634 ± 0.000709	0.994639 ± 0.002824	0.996590 ± 0.001099
LSTM	0.994553 ± 0.001673	0.999313 ± 0.001449	0.995862 ± 0.006039	0.994957 ± 0.001740
GRU	0.999885 ± 0.000160	0.999885 ± 0.000160	0.999885 ± 0.000160	0.999885 ± 0.000160

**Table 15:** Wilcoxon signed-rank test results for online retail dataset (ROC-AUC).

Model pair	P-value	Significance
LSTM versus GRU	1.0000	No
LSTM versus logistic regression	0.0078	Yes
LSTM versus SVM	0.0020	Yes
GRU versus logistic regression	0.0078	Yes
GRU versus SVM	0.0020	Yes
Logistic regression versus SVM	0.0645	No

Also, a comprehensive evaluation of model performance on the Events dataset was conducted using both descriptive statistics and statistical significance testing.The mean and standard deviation results (Table 16) indicate that all models achieve consistently high performance across evaluation metrics, with relatively low variability across multiple runs. Among the evaluated models, GRU achieves the highest accuracy and F1-score, while LSTM demonstrates comparable performance with slightly higher variance, suggesting moderate sensitivity to initialization.Traditional models, including Logistic Regression and SVM, also exhibit competitive performance, particularly in terms of recall. However, their precision and F1-scores are slightly lower compared to deep learning models, indicating a less balanced performance in capturing churn behavior.To further assess whether these observed differences are statistically significant, pairwise Wilcoxon signed-rank tests were performed based on ROC-AUC scores (Table 17). The results show that LSTM significantly outperforms Logistic Regression (p < 0.05), while no statistically significant differences are observed between most other model pairs, including LSTM vs GRU and GRU vs SVM (p > 0.05). This indicates that performance differences across models are generally small and not statistically robust.Overall, the combined analysis of mean performance, variability, and statistical significance suggests that although deep learning models achieve slightly better results, the differences between models on the Events dataset remain limited. This behavior reflects the inherent complexity and noisier nature of event-based data, where model performance tends to converge despite differences in model architecture.

**Table 16:** Mean and standard deviation of model performance over 10 runs (events dataset).

Model	Accuracy	Precision	Recall	F1-score
Logistic regression	0.995447 ± 0.000837	0.988773 ± 0.002039	1.000000 ± 0.000000	0.994354 ± 0.001032
SVM	0.995471 ± 0.001118	0.989340 ± 0.002174	0.999473 ± 0.000802	0.994380 ± 0.001383
LSTM	0.996222 ± 0.002314	0.992591 ± 0.006377	0.998068 ± 0.002515	0.995308 ± 0.002860
GRU	0.996949 ± 0.001998	0.996162 ± 0.004630	0.996253 ± 0.004105	0.996196 ± 0.002487

**Table 17:** Wilcoxon signed-rank test results for events dataset (ROC-AUC).

Model pair	P-value	Significance
LSTM versus GRU	0.9102	No
LSTM versus logistic regression	0.0273	Yes
LSTM versus SVM	0.6953	No
GRU versus logistic regression	0.0781	No
GRU versus SVM	0.6250	No
Logistic regression versus SVM	0.0020	Yes

Overall, the comparative analysis across the two datasets highlights the impact of data characteristics on model performance and statistical behavior.The Online Retail dataset, with its structured and informative RFM features, enables clearer model differentiation and more statistically significant improvements. In contrast, the Events dataset presents a more challenging scenario due to its implicit and noisy behavioral signals, resulting in closer performance across models and fewer statistically significant differences.These observations emphasize that performance gains are not solely dependent on model architecture, but are strongly influenced by the quality and representation of input features. In this context, the integration of Deep Embedded Clustering (DEC) plays a critical role in enhancing feature representation, contributing to stable and competitive performance across both structured and complex datasets.

### Comparison between online retail and events datasets

A comparative analysis was conducted to evaluate model performance across the Online Retail and Events datasets. The results indicates that all models achieve higher performance on the Online Retail dataset.This difference can be attributed to the structured nature of the Online Retail dataset, where customer behavior is explicitly represented through RFM features (Recency, Frequency, and Monetary value), providing clear and informative signals for churn prediction.In contrast, the Events dataset consists of implicit user interactions such as clicks, views, and session activities, which introduce higher levels of noise and behavioral variability. Consequently, modeling customer behavior in this dataset becomes more challenging, leading to relatively lower predictive performance.Despite this complexity, the integration of Deep Embedded Clustering (DEC) plays a crucial role in enhancing feature representation. By transforming raw behavioral patterns into more separable latent representations, DEC improves cluster structure and facilitates more effective learning, particularly in the Events dataset.Deep learning models (LSTM and GRU) demonstrate strong performance across both datasets. In particular, GRU consistently achieves higher recall in detecting churn cases, highlighting the effectiveness of sequential models in capturing temporal dependencies.Furthermore, confusion matrix analysis reveals that misclassification is more frequent in the Events dataset, further confirming its increased complexity. However, the performance gap between the two datasets is reduced when using the proposed framework, indicating the effectiveness of DEC in handling noisy and unstructured behavioral data.Overall, this comparison highlights that while the Online Retail dataset provides a more favorable environment due to its structured representation, the proposed framework remains effective across both structured and complex datasets, demonstrating its robustness and general applicability.

## Comparison with related work

To evaluate the effectiveness of the proposed framework, a comparison is conducted with several recent studies on customer churn prediction using different datasets. Existing approaches primarily rely on traditional machine learning models such as Random Forest and boosting techniques, which achieve strong performance when combined with carefully defined churn criteria. However, these methods depend heavily on handcrafted features and lack the ability to capture deep behavioral patterns. More recent studies have explored deep learning approaches, achieving improved performance by modeling complex customer behavior. Nevertheless, these approaches often do not incorporate structured segmentation or clustering mechanisms. In contrast, the proposed framework integrates RFM-based feature engineering, deep embedded clustering (DEC), and sequential modeling using LSTM and GRU. This unified approach enables more accurate representation of customer behavior and improves predictive performance across different datasets.

**Table 18:** Comparison of the proposed approach with existing churn prediction methods.

Reference	Year	Dataset	Churn definition	Methods and results
Wu et al.^38^	2022	Olist	Customers inactive for 6 months are labeled as churned	PCA-AdaBoost achieved 98.98% accuracy
Baghla and Gupta^39^	2022	Olist	No transactions for 90 days $\rightarrow$ churn	Random Forest achieved 99.35% accuracy
Beckhauser and Filho^40^	2023	Olist	Inactive for 90 days $\rightarrow$ churn	Random forest achieved 89.56% accuracy
Chen et al.^41^	2025	Telecom	Service cancellation	Deep neural networks accuracy = 82.26% ,F1 Score = 82.09%
Proposed work	-	Online retail	Inactive for 90 days $\rightarrow$ churn	LSTM: Acc = 99.65%, F1 = 99.48%
				GRU: Acc = 99.77%, F1 = 99.66%
Proposed Work	-	Events	Inactive for 90 days $\rightarrow$ churn	LSTM: Acc = 99.83%, F1 = 99.78%
				GRU: Acc = 99.75%, F1 = 99.68%

Table 18 presents a comparison between the proposed framework and existing churn prediction approaches across different datasets and churn definitions. The results clearly demonstrate that the proposed approach outperforms prior studies in terms of predictive performance. For instance, Wu et al. (2022) achieved an accuracy of 98.98% using PCA-AdaBoost, while Baghla and Gupta (2022) reported 99.35% accuracy using Random Forest. However, the proposed model achieves higher performance, reaching up to 99.83% accuracy. Similarly, Beckhauser and Filho (2023) reported a lower accuracy of 89.56% using Random Forest, highlighting the limitations of traditional machine learning models when dealing with complex customer behavior patterns. In contrast, the proposed deep learning-based framework consistently achieves near-perfect results across both datasets. Furthermore, Chen et al. (2025) applied deep neural networks on telecom data and achieved an accuracy of 82.26% and F1-score of 82.09%, which is significantly lower than the performance obtained by the proposed approach. This difference can be attributed to the integration of RFM-based feature engineering and Deep Embedded Clustering (DEC), which enhances the representation of customer behavior and improves class separability. It is also important to note that variations in datasets and churn definitions may affect performance comparisons. Nevertheless, the proposed framework demonstrates strong generalization capability and comparable performance across different scenarios.Overall, the results confirm that combining behavioral features with representation learning techniques provides a more effective solution for customer churn prediction compared to existing methods.

## Limitations of the proposed framework

Despite the strong performance achieved by the proposed hybrid framework, several limitations should be acknowledged. First, the Deep Embedded Clustering (DEC) model introduces additional computational complexity during training. Although it improves feature representation and cluster separability, it increases the overall training time compared to traditional clustering methods. Second, the proposed approach depends on predefined thresholds for churn labeling (90 days of inactivity). This threshold may not generalize optimally across different domains or industries, where customer behavior patterns may vary significantly. Finally, although the framework demonstrates strong generalization across two datasets, further validation on larger and more diverse datasets is required to confirm its scalability and adaptability in real-world applications.

## Conclusion

This paper presented a hybrid deep learning framework for customer churn prediction by integrating RFM-based feature engineering with Deep Embedded Clustering (DEC) and sequential deep learning models.The experimental results on both Online Retail and Events datasets demonstrate that the proposed framework achieves consistently high performance across multiple evaluation metrics.Statistical analysis based on 10 independent runs and Wilcoxon signed-rank tests confirms the robustness of the results and shows that deep learning models significantly outperform traditional approaches, while no significant difference is observed between LSTM and GRU.The findings highlight that performance improvements are primarily driven by enhanced feature representation rather than model architecture alone. In particular, the integration of DEC plays a critical role in transforming customer behavior into more separable latent representations, which improves model effectiveness, especially in complex and noisy datasets such as the Events dataset.Furthermore, the comparative analysis between datasets shows that while structured transactional data (Online Retail) leads to higher predictive performance, the proposed framework remains effective in handling more challenging, real-world behavioral data.Overall, the proposed approach demonstrates strong robustness, generalization capability, and practical applicability for customer churn prediction across different types of datasets.

## Future work

Despite the strong performance of the proposed framework, several directions can be explored for future research. First, adaptive and data-driven approaches for churn labeling can be investigated to replace fixed thresholds. Such approaches would allow the model to better generalize across different domains and customer behavior patterns. Second, the proposed framework can be evaluated on larger and more diverse real-world datasets to further assess its scalability, robustness, and applicability in practical business environments. Third, advanced deep learning architectures, such as attention-based and transformer models, can be explored in combination with DEC-based feature representation to further enhance sequential modeling of customer behavior. Finally, future work may investigate integrating additional behavioral and contextual features to further enhance representation learning and improve prediction performance.

## Data Availability

All datasets are freely available, and their source is cited.
